# Implementation of a Modified Fracture Liaison Service at Aarhus University Hospital: A 2-Year Retrospective Cohort Study

**DOI:** 10.1007/s00223-026-01478-x

**Published:** 2026-01-23

**Authors:** Vivi-Nelli Mäkinen, Anne Sophie Sølling, Torben Harsløf, Bente L. Langdahl

**Affiliations:** 1 Department of Endocrinology and Internal Medicine, Aarhus University Hospital, Aarhus, Denmark; 2 Department of Clinical Medicine, Aarhus University, Aarhus, Denmark

**Keywords:** Fracture liaison service, FLS, Fragility fracture, Osteoporosis, Prevention

## Abstract

Fragility fractures are a consequence of osteoporosis and the strongest indicator of sustaining a future fracture. Effective fracture prevention strategies are therefore needed to reduce the incidence of fragility fractures. Fracture Liaison Services (FLS) are globally recognized as the most effective approach. Inspired by their success, the Danish Health Authority recommended nationwide implementation of prevention programs. With limited funding, the fracture prevention program at Aarhus University Hospital (AUH) was established in 2022 as a modified version. Here, we describe the implementation with focus on patient capture, treatment uptake, collaboration with primary care physicians, and implementation challenges. A retrospective single-center cohort study was conducted. We retrieved information from the hospital registries. Eligible patients were those ≥ 50 years of age who sustained a fracture of the hip, pelvis, spine, distal forearm, or shoulder. Twenty-four months after the implementation, 2363 patients with a fracture were identified. Excluding patients not eligible for the program (n = 1096, 46%), 890 DXAs were performed. Patients diagnosed with osteoporosis following their attendance in the program accounted for 22% (n = 198). Sixty-five percent were potentially eligible for bone anabolic treatment. The fracture prevention program has systematized the identification of patients presenting with a fragility fracture, and identified osteoporosis in 22% of the patients, and ensured a successful collaboration with the primary care physicians. Despite lacking some components of a full FLS, we have demonstrated that it is possible to successfully establish an FLS modified according to available resources and identify patients at high risk of future fractures.

**Supplementary Information:**

The online version contains supplementary material available at 10.1007/s00223-026-01478-x.

## Introduction

Fragility fractures are the major clinical complication of osteoporosis, and as the global population ages, the number of people living with osteoporosis and sustaining fragility fractures is expected to increase [1, 2]. These fractures significantly impact both physical and mental health, leading to loss of independence, impaired quality of life, and higher morbidity and mortality [3]. Multiple factors contribute to the risk of sustaining a fragility fracture, however, one of the strongest indicators for a high fracture risk in the near future is a recent fracture. This “imminent fracture risk” remains for 12–24 months [4]. Despite this knowledge, a global care gap remains in the diagnosis and management of osteoporosis among fracture patients, and less than 20% of these patients receive pharmacologic treatment (3) [5, 6].

Establishing the original Fracture Liaison Service (FLS) in Glasgow, Scotland, became a cornerstone in improving secondary fracture management through identifying, evaluating, and treating osteoporosis [7]. The value of FLS has since been widely recognized as a successful, cost-effective solution to address this care gap and prevent secondary fractures [8, 9]. However, effective fracture prevention is hindered by undertreatment and poor adherence [10]. At the same time, surveys show that primary care physicians (PCP) lack practical guidance on osteoporosis management and are hesitant to prescribe treatment, leading to low participation in secondary fracture prevention. Addressing this gap requires clear information on available antiosteoporosis treatments and optimal management [11, 12].

Scandinavian countries are among those with the highest incidence of fractures. In Denmark, only one in three of the 650,000 individuals estimated to have osteoporosis is diagnosed [2, 13, 14]. In 2018, the Danish Health Authorities recommended the implementation of fracture prevention programs at all Danish hospitals to optimize the management of individuals who sustain fragility fractures [15]. Adapted to the reality of available resources, a tailored model of the FLS was implemented at Aarhus University Hospital (AUH), Denmark, in May 2022.

This study aims to describe our experiences with implementing a modified FLS at a university hospital in Denmark, focusing on participation in the FLS program, the uptake of the secondary fracture prevention, including collaboration with the PCPs, and the challenges associated with the implementation.

## Material and Methods

### Study Design

We conducted a retrospective 2-year single-center cohort study at AUH. All patients aged 50 years or older presenting with a major osteoporotic fracture (hip, spine, forearm, shoulder) or fracture of the pelvis between 1st of May 2022 and 30th of April 2024, were assessed. Patients were identified using the Electronic Patient Record (EPR) system. A manual case-by-case review was performed, and information about demographic factors, trauma mechanism, and results from previous the bone health assessment was collected.

### Setting–FLS at Aarhus University Hospital

AUH is an academic tertiary care referral center linked to 125 primary care centers with 230 PCPs, and provides health coverage to approximately 365,000 citizens [16, 17]. Before the initiation of our program, there was no flagging system for patients with fractures. The fracture prevention program at AUH is an adapted model of the FLS tailored to the available resources. Therefore, we decided to focus on fractures that would qualify the patients for bone anabolic treatment according to Danish reimbursement criteria (supplementary appendix 1).The FLS team consists of four trained nurse practitioners supported by four osteoporosis specialists. All clinical personnel involved in FLS contribute to the program alongside their other responsibilities at the endocrinology outpatient unit.

The FLS AUH program consists of several phases of activities. The key aspects are:

i. Capture system.

**Fig. 1:**
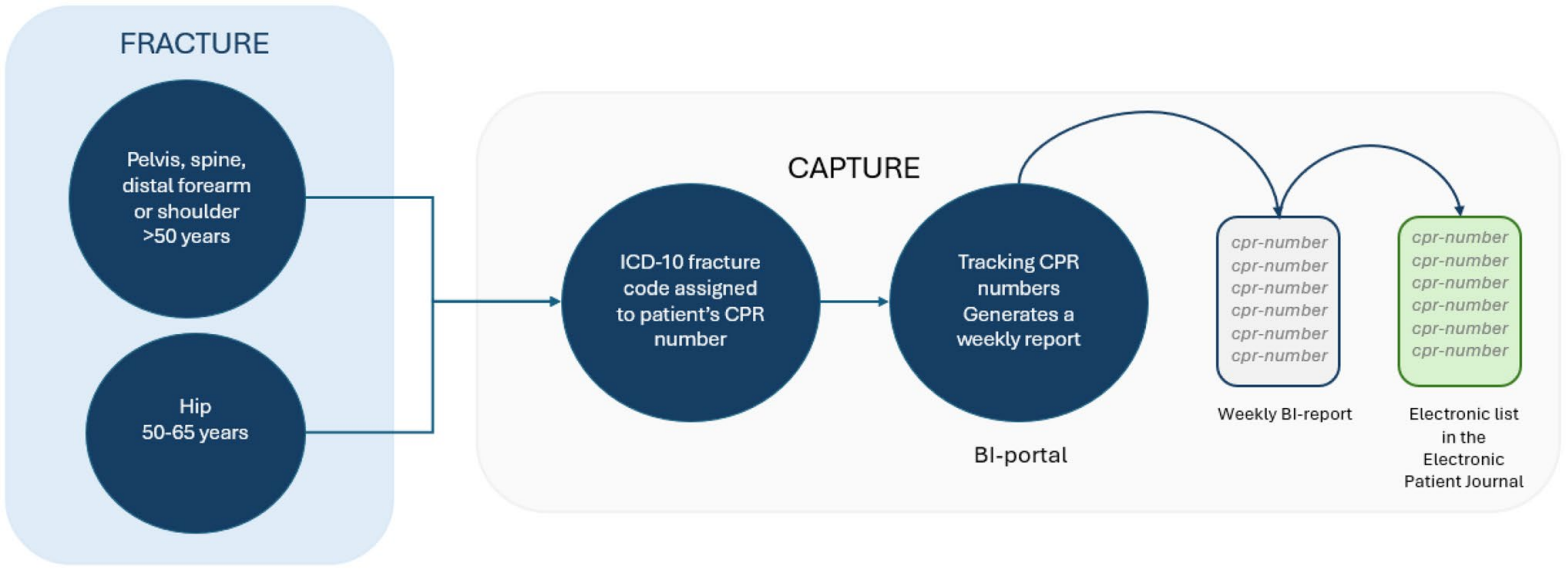
Overview of the capture system at Aarhus University Hospital. The CPR numbers of patients aged 50 years or older, treated at the emergency department for a relevant fracture, are automatically added to a list. These CPR numbers are then manually transferred to a list within the Electronic Patient Record system, enabling the nurse practitioner to review the corresponding medical records manually

Each citizen in Denmark has a unique civil registration number (CPR) [18]. Each week, an automated list of patients aged 50 years or older is generated, comprising those who have received a diagnostic code for a fracture from the Emergency Department at AUH. The patients’ CPR numbers are then manually transferred to an electronic list in the EPR system, which is reconciled by the nurse practitioners (Fig. 1).

ii. Patient evaluation.

The initial post-fracture assessment is led by a trained nurse practitioner, who follows pre-agreed protocols (supplementary appendix, Fig S1a and b). Patients who sustain a pathological or traumatic fracture, reside outside the hospital district, or have a wrong ICD-10 code are not considered eligible for the program. Patients over 65 years who suffer a hip fracture are not included in the FLS, as they are assessed by the Department of Geriatrics at AUH. Patients already referred to a DXA by their physician or PCP are also excluded.

Moreover, patients with severe comorbidity, cognitive deficiency, terminal malignancy, who are bed-bound, or have an underlying bone disease other than osteoporosis, are after initial evaluation based on hospital records triaged and not eligible for further assessment within the FLS program. This also applies to patients with a DXA with BMD T-scores > − 2 within 24 months before the fracture. Captured patients already diagnosed with osteoporosis are reassessed and invited to the endocrinology outpatient unit if they fulfill the criteria for bone anabolic treatment.

iii. Investigation and treatment suggestion.

Patients eligible for further assessment are contacted by electronic letter, informing them that a bone fracture may be a sign of underlying osteoporosis and providing details about an upcoming follow-up phone consultation. During the phone consultation, the nurse collects further information about the fracture location and trauma, co-morbidities and pharmacological treatments. The nurse also informs the patient about the association between a fragility fracture and osteoporosis. If the patient is interested, a DXA and a vertebral fracture assessment (VFA) are arranged as the initial assessment as part of the phone consultation. An osteoporosis specialist subsequently evaluates the DXA and VFA. A vertebral fracture is defined as a vertebral height loss of > 20%.

The results are forwarded to the patient and their PCP.

After reviewing the information available and the results of DXA and VFA, the specialist may either:

i. Refer the patient directly to the outpatient unit for initiation of bone anabolic treatment, if no contradictions for bone anabolic treatment are found in the hospitals EPR system. In this case, the PCP is informed that the patient has been diagnosed with osteoporosis and treatment will be initiated at the outpatient unit.
ii. Refer the patient to the PCP for initiation of anti-resorptive treatment if osteoporosis is diagnosed, but the patient does not fulfill criteria for bone anabolic treatment, or follow-up in case of osteopenia.
iii. In cases when the specialist cannot determine eligibility for bone anabolic treatment based on the available information in the EPR system, the patient is referred to the PCP to clarify key aspects of the medical history (i.e. cardiovascular risk factors). Once eligibility is established, the PCP can then refer the patient directly to the osteoporosis outpatient unit for initiation of bone anabolic treatment.

The PCP receives written information about further investigations and treatment recommendations according to national guidelines, including indications and contraindications for bone anabolic treatments.

In addition to medical treatment, all patients are recommended an adequate daily intake of calcium and vitamin D, weight-bearing exercise, as well as smoking cessation.

### Statistical Analysis

We summarized data using descriptive statistics or frequencies. Descriptive statistics are presented as mean ± standard deviation (SD) or interquartile range (IQR) in case of violence of normality. Non-parametric testing was conducted if the data distribution did not fulfill the assumptions for normality. All tests were two-sided, and *p*-value of < 0.05 was statistically significant. All analyses were performed using IBM SPSS Statistics version 29.0.1.0 (Statistical Package for the Social Sciences, SPSS Inc., Chicago, Ill., USA).

## Results

### Capture

**Fig. 2:**
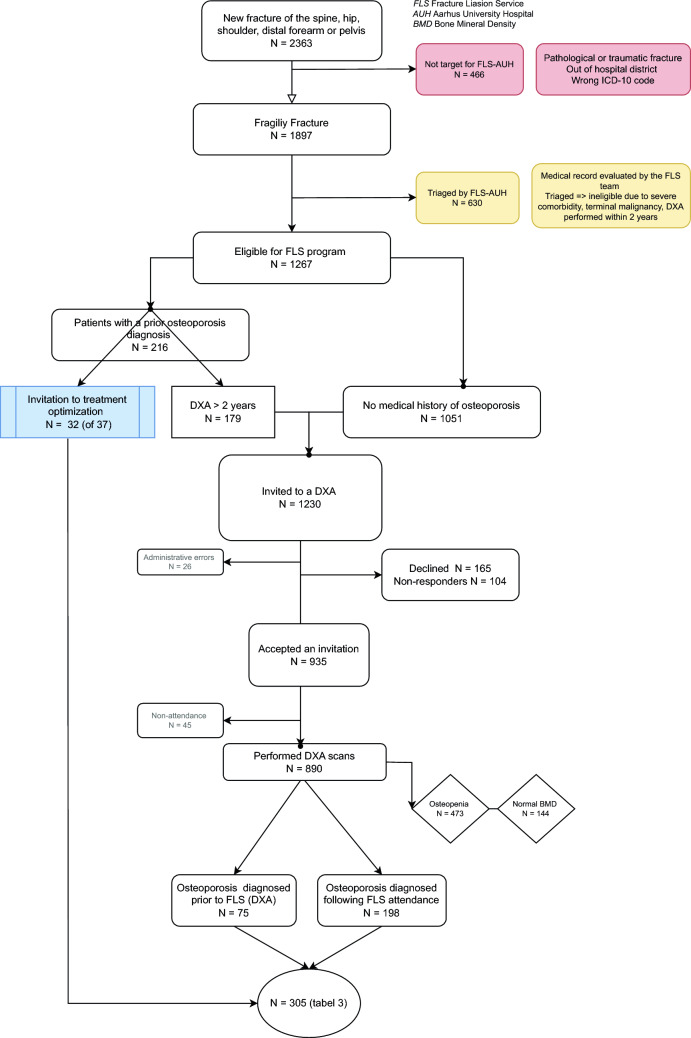
Flow diagram of the 2363 patients captured by the Fracture LiaisonService program at Aarhus University Hospital. Patients highlighted in red and yellow boxes weretriaged and not found eligible for further bone health assessment. Those with a priordiagnosis of osteoporosis were either invited for treatment optimization orscheduled for a new DXA scan as the initial step. For patients without a known history ofosteoporosis, the first step is a DXA scan. After excluding patients who declined, did not respond, orfailed to attend, a total of 890 DXA scans were performed over the 2-year period

Figure 2 displays the flow of patients captured in our FLS. Over the 2 years, 2363 patients sustaining a fracture of the hip, pelvis, spine, forearm, or shoulder were identified and their records reviewed by the coordinators. First, a total of 484 patients (21%) were excluded due to the trauma mechanism or because they were residing outside the hospital district. Following, further 630 patients were triaged and found ineligible due to e.g. terminal illness or severe co-morbidity, making osteoporosis investigation irrelevant or difficult through an FLS setup. Other reasons included a prior diagnosis of osteoporosis with ongoing appropriate treatment, referral for DXA by the primary care physician, or having undergone a DXA within the past 2 years with T-scores greater than − 2.0 (see Table 1 for a more detailed description of these patients).

**Tabel 1:** Patients evaluated by the FLS team and not eligible for DXA or further osteoporosis evaluation in the FLS Setting, n (%)

	Patients (n = 1096)
Triaged patients with a fragility fracture	N = 612 (56%)
Cognitive deficiency	86 (14%)
Malignancy	52 (8%)
Severe comorbidity	25 (4%)
Deceased	2 (0.3%)
Other metabolic bone disorder	2 (0.3%)
Ongoing antiosteoporotic treatment	208 (34%)
Referred to DXA by PCP or specialist	111 (18%)
DXA within two years with normal BMD T-scores or osteopenia	96 (16%)
PCP advised on treatment	26 (4.2%)
Admitted at AUH	4 (0.6%)

Not target for AUH FLS	N = 466 (42%)
Pathological or traumatic fracture	366 (78%)
Outside of hospital district	15 (4%)
No fracture or fracture type not targeted by FLS	85 (18%)

Other	N = 18 (2%)
Unknown	6 (33%)
Other	12 (67%)

*FLS*: Fracture liaison service *AUH*: Aarhus University Hospital *PCP*: Primary care physician.

Of the remaining 1267 consecutive patients with a fragility fracture eligible for the FLS program 935 (74%) accepted an invitation to a DXA. A total of 269 patients (21%) declined or did not respond to the invitation. Based on a DXA within the last 2 years showing osteoporosis, 32 (of 37) patients accepted an invitation for treatment optimization in the outpatient unit. The median time from the index fracture to their first contact with FLS was 26 days (IQR 20;36). From the telephone consultation to a DXA, the median time was 32 days (IQR 32;47). By April 30, 2024, 70% of eligible patients had had a DXA performed.

### Baseline Characteristics

During the 2 years, 890 patients with a new fragility fracture had a DXA performed as part of the FLS program. Patients who did not attend (n = 45) were excluded. A total of 16% had normal BMD (n = 144) and 53% had osteopenia (n = 473).

Baseline characteristics of patients with osteoporosis (the newly diagnosed patients, patients with DXA > 2 years ago, and patients invited for treatment optimization) are shown in Table 2. T-scores presented in Table 2 only accounts for the patients, who had a DXA performed (n = 273).

**Table 2:** Characteristics of all patients with osteoporosis

Characteristics	Patients (n = 305)
Female gender, n (%)	264 (87%)
Age, years (mean, SD)	70 (9.2)
*Fracture site* n, (%)	
Vertebra	21 (7%)
Forearm	180 (59%)
Hip	13 (4%)
Pelvis	15 (5%)
Humerus	76 (25%)
Vertebral fracture(s) identified on VFA, n (%)	46 (16%)
Femoral neck BMD T-score (median, IQR)*	− 2.5 (− 3.0;− 2.0)
Total hip BMD T-score (median, IQR) *	− 2.1 (− 2.5;− 1.5)
Lumbar Spine BMD T-score (median IQR) *	− 2.5 (− 2.9;− 1.4)
BMI < 18.5, n (%)	11 (4%)
18.5–24.9, n (%)	139 (49%)
25–29.9, n (%)	85 (30%)
30–34.9, n (%)	32 (11%)
35–39.9, n (%)	4 (1%)
BMI>40, n (%)	4 (1%)
Vitamin D (mean, SD) nmol/l	107 (35.57)
Vitamin D deficiency,<50 nmol/l, n (%)	13 (4%)
eGFR (mean, SD) ml/min	81 (12.38)
Hemoglobin (mean, SD) mmol/L	8.4 (0.86)
Alkaline phosphate (mean, SD) U/L	82 (30.03)
Smoking, active, n (%)	31 (10%)
Type 2 diabetes, n (%)	18 (6%)
Hypertension, n (%)	93 (30%)
Chronic kidney disease, n (%)	1 (0.3%)
Hyperparathyroidism, n (%)	6 (0.9%)
Hypercholesterolemia, n (%)	74 (24%)
Rheumatoid Arthritis, n (%)	7 (0.2%)

^*^T-scores presented only accounts for the 273 patients who had a DXA performed

*BMD* Bone Mineral Density, *BMI* Body Mass Index, *eGFR* Estimated Glomerular Filtration Rate, *IQR* Interquartile Range, *SD* Standard Deviation, *VFA* Vertebral Fracture Assessment.

The majority of the patients with osteoporosis were women (87%) with a mean age of 70 years (SD 9.18). The most common fracture location was the distal forearm (59%), followed by proximal humerus (25%) and fractures of the vertebrae (7%). Forty-six patients with a non-vertebral fracture were found to have a vertebral fracture identified on the VFA, defined as a vertebral body height loss of > 20%. Ten of these patients had T-scores above − 2.5.

Of the 890 patients attending a DXA, 198 were diagnosed with osteoporosis after participating in the FLS program (Table 3). This includes patients with T-scores in the osteopenic range but who suffered a fragility fracture of the hip or spine. 473 patients had osteopenia without vertebral or hip fracture. According to Danish guidelines these patients are not offered treatment unless they are treated chronically with glucocorticoids.

**Table 3:** Characteristics of patients diagnosed with osteoporosis in FLS^a^

Characteristics	Patients (n = 198)
Female gender, n (%)	164 (83%)
Age, years (mean, SD)	69.30 (9.18)
*Fracture site* (n, %)	
Vertebra	14 (7%)
Forearm	117 (59%)
Hip	9 (5%)
Pelvis	7 (4%)
Humerus/shoulder	51 (26%)
Vertebral fracture(s) identified on VFA, n (%)	27 (14%)
Femoral neck T-score (median, IQR)	− 2.5 (− 2.1; − 2.9)
Total hip T-score (median, IQR)	− 2.1 (− 1.5; − 2.5)
Lumbar spine T-score (median IQR)	− 2.5 (− 1.4; − 3.1)
Vitamin D (nmol/l) (mean, SD)	103 (34)
Vitamin D deficiency (< 50 nmol/l), n (%)	10 (5%)
Smoking, active, n (%)	23 (17%)

^a^Defined as patients with no previous ICD-10 code for osteoporosis in their medical journal

FLS: Fracture liaison service IQR: Interquartile range VFA: Vertebral fracture assessment SD: Standard deviation.

The majority were women (n = 165, 83%), and the mean age was 69 years (SD 9.16). A fracture of the distal radius was the most common type. By the 30th of September 2024, treatment was prescribed for the majority of the newly diagnosed patients (n = 171, 85%). The median time from the DXA to treatment initiation was 57 days (IQR 35;97).

One hundred sixty-five patients (13%) declined the service, providing the primary reasons for declining as (i) lack of interest in the program and (ii) inability to attend due to physical limitations. Compared to FLS attenders, these patients were older (77 years versus 70 years, p < 0.001) and more frequently men (21% vs. 13%, p = 0.032). The number of individuals with previous fragility fractures was balanced between the two groups (15% decliners versus 16% attenders). According to the medical records, 44 of the patients declining the service had been diagnosed with osteoporosis before FLS capture. A large percentage of these patients with osteoporosis did not receive treatment at the time of their index fracture (43%).

### Treatment at the Endocrinological Outpatient Unit

#### Patients with Previously Unrecognized Osteoporosis

**Fig. 3:**
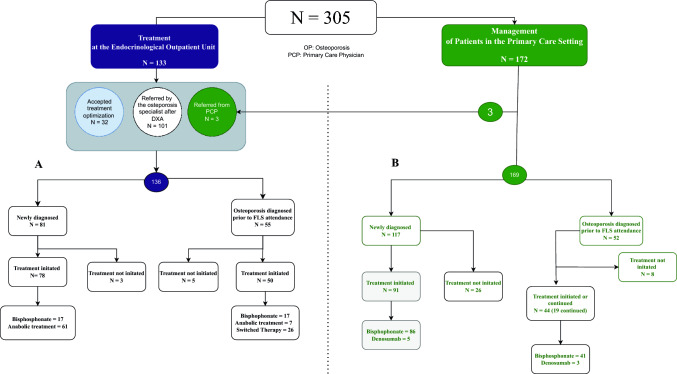
Treatment initiation status for 305 patients, categorized by site ofinitiation—either the Endocrinology Outpatient Clinic (**A**) or the patient’sprimary care physician (**B)**—and by whether the patient had a priordiagnosis of osteoporosis before attending the FLS program. Three patients werereferred to the outpatient unit by their PCP

Figure. 3 shows treatment flow of patients with osteoporosis. Eighty-one newly diagnosed patients were directly referred to the outpatient clinic. In 61 cases, bone anabolic treatment with romosozumab or teriparatide was initiated (Fig. 3A). For the remaining patients (n = 17), an antiresorptive drug was opted for. Five patients preferred alendronate due to personal reasons, such as fear of adverse events. The treating endocrinologist opted for bisphosphonates due to the patient’s cardiovascular risk factors or history of stroke or acute myocardial infarction in six cases. Three patients declined any treatment.

Screening for secondary causes of osteoporosis prompted a referral to further diagnostic investigation for primary hyperparathyroidism or hematological disease in four patients. In the meantime, these patients were treated with an infusion of zoledronate.

#### Captured Patients with Prior Osteoporosis Diagnosis

A total of 55 patients diagnosed with osteoporosis before their index fracture were referred to the outpatient clinic. The patients were either referred by their PCP (n = 3) or the osteoporosis specialist (n = 52), who had reviewed the patient’s DXA (Fig. 3A).

From the initial 55 referred patients, 33 (61%) were prescribed romosozumab or teriparatide and 17 (30%) antiresorptives. Two patients preferred bisphosphonates due to personal reasons, such as fear of adverse events. In the remaining cases (n = 15), the treating endocrinologist opted for bisphosphonates due to the patient’s cardiovascular risk factors or history of stroke or acute myocardial infarction. Five patients declined treatment initiation.

### Management of Patients in the Primary Care Setting

#### Patients with Previously Unrecognized Osteoporosis

One hundred and seventeen patients were initially referred to the PCP for treatment initiation (Fig. 3B). In 49 cases, the PCP was advised to consider the patient’s eligibility for anabolic treatment and make a referral to the outpatient unit if the criteria were fulfilled and the patient was interested. Only three were referred back to the endocrinological outpatient clinic. The remaining non-referred newly diagnosed patients were started on an oral bisphosphonate or denosumab. Treatment was not initiated in 26 (23%) patients by September 30, 2024. Screening for secondary causes of osteoporosis was performed in 80% of patients before treatment started (blood samples within three months).

Fifty-two patients were already diagnosed with osteoporosis before being captured by the FLS service. The majority of these patients continued or had an oral bisphosphonate prescribed by their PCP. Eight patients did not receive any osteoporosis treatment either before or after their participation in the FLS program.

## Discussion

This is the first comprehensive study evaluating the implementation of a FLS program in Denmark over a 2-year period. In line with other studies, the FLS-AUH program has demonstrated the need for a systematic approach to improve osteoporosis management of fracture patients.

Our study shows several benefits of implementing a modified FLS program within an established outpatient unit. One of the most notable is the efficacy of reducing the treatment gap in PCP administrated treatments. As of the 30th of September 2024, 78% of the patients referred to the PCPs for further investigations and treatment initiation were prescribed antiosteoporosis treatment. Compared to multiple studies reporting markedly lower treatment rates (15–70%), our results demonstrate how the program facilitates efficient communication and thereby treatment initiation [19–25]. Based on a comprehensive review of treatment rates in FLS settings across 46 studies by Wu et al., we assume that treatment is predominantly initiated within the framework of the FLS [26]. Our data highlights the potential of PCPs to act as a resource in ensuring treatment initiation, especially when providing them with the needed information.

Secondly, by performing a VFA, we identified vertebral fractures in 46 patients who presented with a non-vertebral fracture. This rate (15%) is comparable to rates reported by Binkley et al. [27]. This emphasizes the value of including VFA in general, but particularly in patients with peripheral fractures, as information about vertebral fractures may assist in therapeutic decision-making [27, 28].

During the 2 years, the program captured 2632 fracture patients. After excluding ineligible patients and triaging patients previously diagnosed with osteoporosis and patients with a very high co-morbidity burden, the response rate was 70% among the 1267 patients with a recent fragility fracture invited for a DXA or treatment optimization. Compared to previous studies reporting attendance rates between 20 and 86%, our response rate of 70% is consistent with the higher end of the spectrum, confirming that an FLS is a significant step forward in the secondary prevention of subsequent fractures in fracture patients [29, 30].

A key strength of our program was the manual, case-by-case review of medical records prior to inviting patients to the FLS program. Although time-consuming, this approach allowed us to identify and exclude 484 patients who did not meet the eligibility criteria—most commonly due to traumatic fractures and further triage 630 patients already diagnosed with osteoporosis or in whom the co-morbidity burden was very high (Table 1). By doing so, we prevented unnecessary DXA invitations, ultimately reducing the cost of performing DXAs and thereby optimizing the available resources.

During the 2 years, 890 DXAs were performed, and 71% were diagnosed with either osteoporosis (n = 198) or osteopenia (n = 473). Our data are comparable to a study by Chevalley et al. which reported 86% to have osteoporosis or osteopenia [31], considering that our program currently does not include patients over the age of 65 with a proximal femur fracture. The Geriatric Department manages these patients who are most often treated with zoledronate.

At the time of the evaluation of their DXA, 182 met the immediate criteria for bone anabolic treatment, however, it was only initiated in 94 cases (61 newly diagnosed and 33 with a prior osteoporosis diagnosis). As bone anabolic treatment is often recommended as the first-line therapy for high-risk patients, it is crucial that we successfully identify and capture these patients [32]. Despite the recommendation of bone anabolic treatment in the DXA report and communication with the PCP, only a limited number of patients were referred to the outpatient unit (n = 3). This highlights the need to improve the information provided to the PCPs, including more details about the benefits of bone anabolic treatment compared to antiresorptive treatment in patients at high risk of fracture. While some patients may have contraindications to bone anabolic treatment, it is unlikely to be the case in as many as 47, suggesting that a better understanding of the reasons for not referring patients for bone anabolic treatment could enable the PCP to become an active stakeholder in the management of these high-risk patients.

Regardless of the efforts of a dedicated FLS team, 165 patients (13%) declined the service, 104 (8%) patients did not respond, and 45 (5%) patients did not show up for DXA. Our over-all attendance rate for DXA was 72% (890 performed DXAs out of 1230 eligible for a DXA invitation). Frequently mentioned reasons for not attending the program included: not interested, physically unable to attend the clinic, or intrinsic motivations. [7, 33]. To further understand this patient group, qualitative studies are necessary to describe these patients’ characteristics. Gaining this information could also increase attendance rates, as it would allow us to identify and eliminate factors associated with declining the service, not responding and patients not showing up to DXA. In the meantime, innovative strategies are encouraged i.e. to engage patients in person during post-fracture care follow-up visits [34]. Moreover, the number of untreated and unmotivated patients highlights the need for increased education about the benefits of secondary fracture prevention to improve attendance rates. In some cases, it may even be beneficial to initiate treatment without performing a DXA as an initial step, especially for frail patients with severe comorbidities and high age.

There are several limitations to our study. First, a comparison of fracture patients offered screening and treatment for osteoporosis before the FLS implementation was not performed. Similarly, we did not evaluate the number of secondary fractures before and after the FLS implementation or the cost-effectiveness. Additionally, information on the reasons for non-attendance and information on patients in whom treatment was not prescribed was not available. Relying solely on ICD-10 coding to identify fragility fractures may have its limitations, as there is a risk of fractures not being coded correctly and therefore missed [35]. Finally, it is also a limitation that not all fracture types recommended being included in an FLS are included in our FLS. Patients with these fracture types could potentially benefit from FLS care, but due to lack of resources, e.g. the limited number of nurse practitioners and the clerical time needed to reviewing patient records, they are currently not included.

## Conclusion

The FLS at AUH has effectively identified patients with fragility fractures who are at a high risk of subsequent fractures. Although there are clear gaps and limitations to our program, we have successfully identified patients with previously unknown osteoporosis, many of whom were at high risk for further fractures and thus eligible for bone anabolic treatment.

## Supplementary Information

Below is the link to the electronic supplementary material.

Supplementary file 1 (DOCX 221 kb).
