# Supplementary material for: Implementation of a Modified Fracture Liaison Service at Aarhus University Hospital: A 2-Year Retrospective Cohort Study
Source: Calcif Tissue Int. 2026 Jan 23;117(1):14. doi: 10.1007/s00223-026-01478-x (PMC12830402; doi:10.1007/s00223-026-01478-x)
Supplement: Supplementary file 1 — (DOCX 221 kb). [file 223_2026_1478_MOESM1_ESM.docx]

Supplementary Appendix 1

**ICD-10 codes**

DM485

DM485A

DM800

DM809

DM809C

DS220

DS220A-L

DS230

DS320A-D

DS328

DS328

DT089

DS323

DS324

DS325

DS327B

DS328B

DS328C

DM809B

DS720

DS721

DS721B

DS722

DS729

DS422

DS422A

DS422B

DS422C

DS423

DS423A

DS424

DS424A

DS424B

DS424

DS428

DS429

DS520

DS520A

DS520B

DS521

DS521A

DS521B

DS522

DS523

DS524

DS525

DS525A

DS525B

DS525C

DS526

DS528

DS528A

DS528B

DS528C

DS529

**Fig S1A** Flowchart showcasing the pre-approved local protocol for eligible FLS patient with a fracture of the humerus, distal forearm or pelvis. Red boxes indicate no further action; blue invitation for a new DXA scan or treatment recommendation; green assessment needed by an osteoporosis specialist


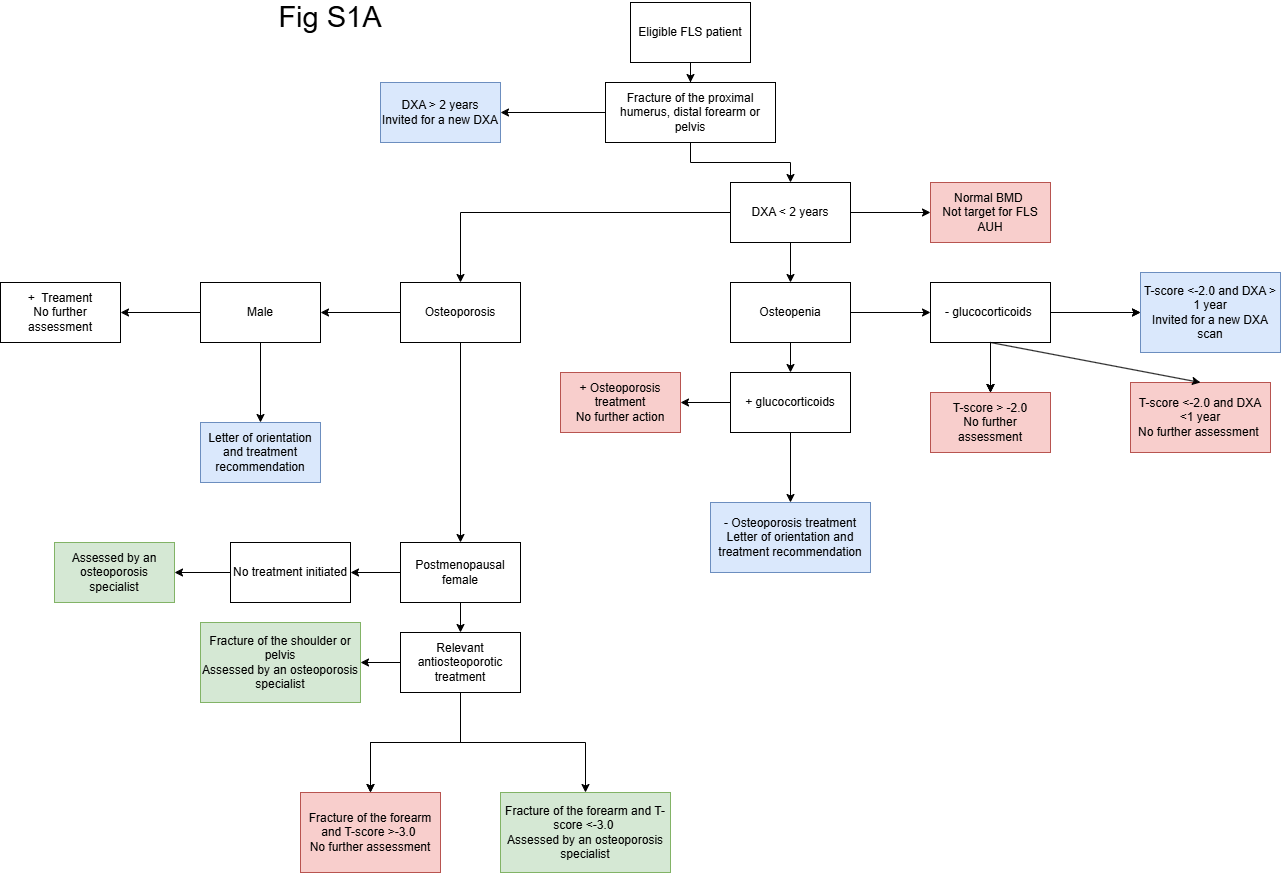


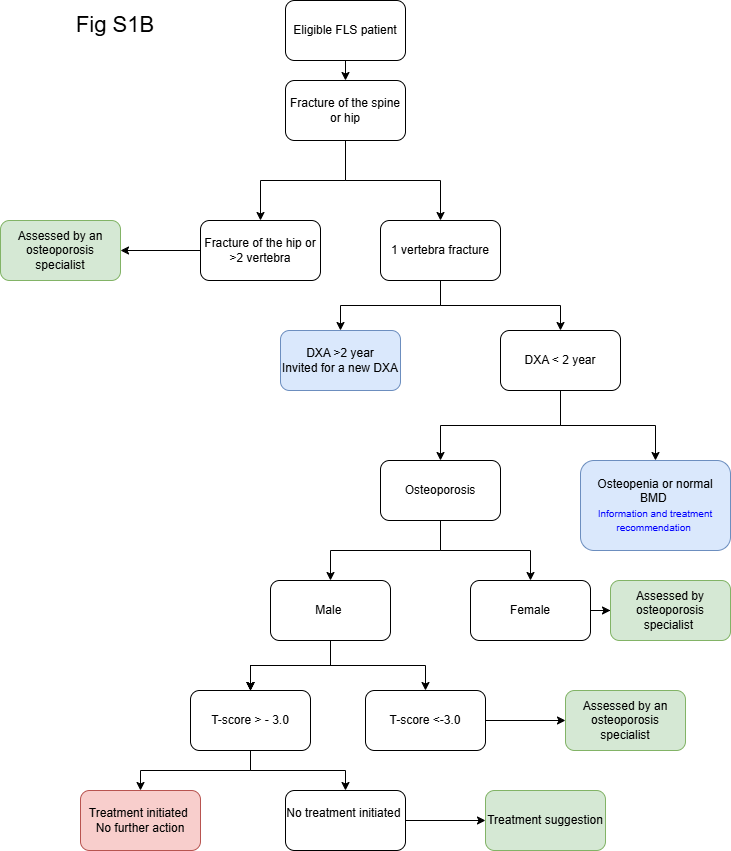


**Fig S1B** Flowchart showcasing the pre-approved local protocol for eligible FLS patient with a fracture of the hip or spine. Red boxes indicate no further action; blue invitation for a new DXA scan or treatment recommendation; green assessment needed by an osteoporosis specialist
